# Myocardial Enhancement Following Agitated Saline Contrast Study in a Boxer Dog

**DOI:** 10.1016/j.case.2023.02.009

**Published:** 2023-04-30

**Authors:** Karen E. Moy-Trigilio, Bruce W. Keene, Piers Barker, Darcy Adin

**Affiliations:** a Department of Small Animal Clinical Sciences, College of Veterinary Medicine, University of Florida, Gainesville, Florida; b Department of Clinical Sciences, College of Veterinary Medicine, North Carolina State University, Raleigh, North Carolina; c Duke Pediatric and Congenital Heart Center, Duke University Medical Center, Durham, North Carolina

**Keywords:** Air embolism, Atrial septal defect, Right-to-left shunt

## Abstract

• Myocardial enhancement after agitated saline contrast study in a dog is described.
• Suspect air microemboli can inadvertently be introduced into coronary vasculature.
• Air microemboli are a theoretic risk of saline contrast echocardiography.

## Graphical abstract

**Figure:**
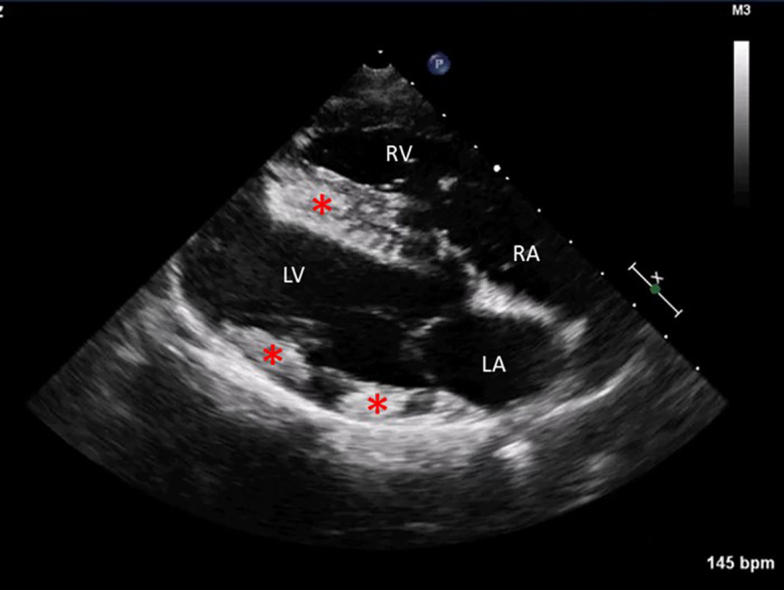

## Introduction

Echocardiography is the primary imaging modality for diagnosing heart disease in animals and people. While color flow Doppler can identify intracardiac blood flow direction and velocity, the injection of agitated saline (introducing microscopic air bubbles to perform a “saline contrast echocardiogram” or “bubblegram”) is additive as it allows for the detection of right-to-left intracardiac or vascular shunts without the need for anesthesia, which is a requirement for advanced imaging modalities such as computed tomography in animals.^1^, ^2^, ^3^, ^4^ The microbubbles introduced into the venous circulation are normally removed from the intravascular space as they pass through the pulmonary capillaries, such that echocardiographic contrast is expected to be visualized only in the right side of the heart or systemic venous circulation but not in the left side of the heart or systemic arterial circulation. Saline contrast echocardiography is therefore considered confirmatory for a right-to-left shunt when microbubbles are detected in the left side of the heart following intravenous injection.^1^, ^2^, ^3^, ^4^, ^5^, ^6^

Although saline contrast echocardiography is routinely performed in veterinary cardiology, we are unaware of reported complications in dogs.^7^ We describe a case of echocardiographic myocardial contrast enhancement following the intravenous administration of agitated saline in a dog with tricuspid dysplasia and an atrial septal defect (ASD).

## Case Presentation

A 9-month-old female spayed 20.1 kg boxer dog was presented to the cardiology service at North Carolina State University College of Veterinary Medicine for evaluation of a 3-month history of collapsing episodes. She was otherwise healthy.

**Figure 1:**
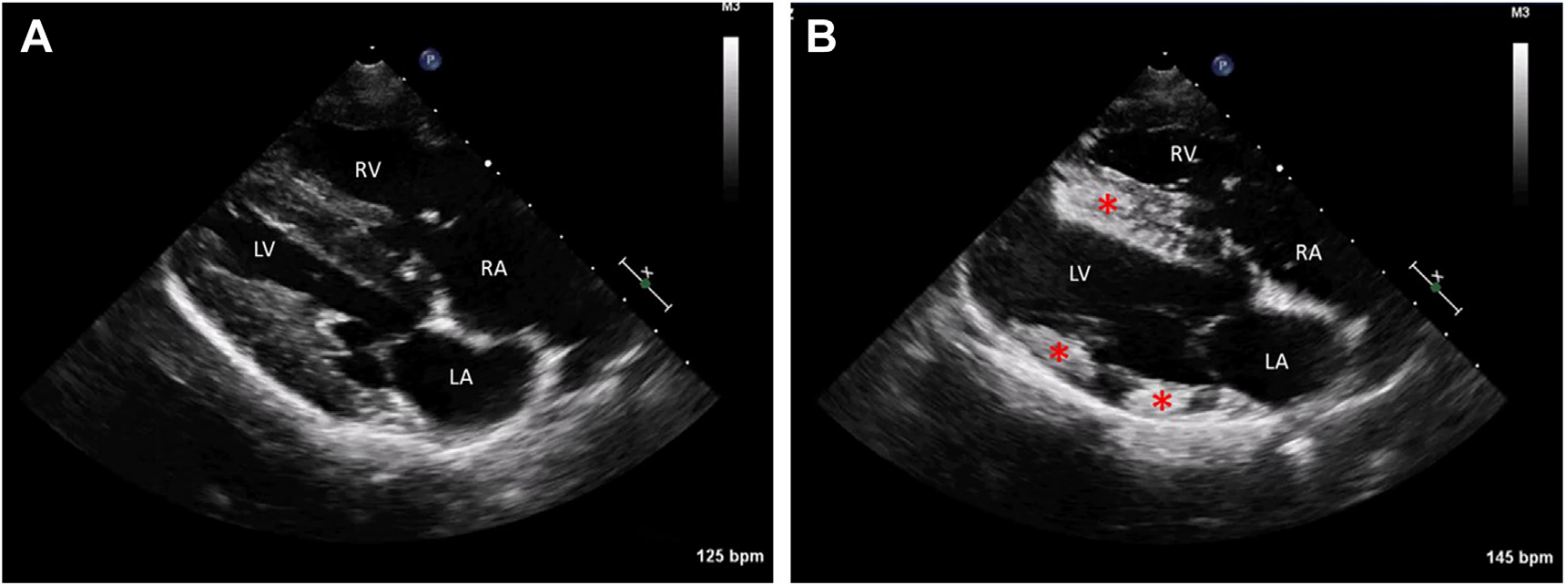
Two-dimensional TTE in the right parasternal 4-chamber long-axis view. The ASD is not visible in this image. **(A)** Normal myocardial echogenicity before the saline contrast echocardiogram was performed. **(B)**. Patchy myocardial enhancement of the interventricular septum and left ventricular free wall, depicted by the *red asterisk* (∗), following saline contrast echocardiography and after microbubbles dissipated from the cardiac chambers. *LA*, Left atrium; *LV*, left ventricle; *RA*, right atrium; *RV*, right ventricle.

On presentation, she was bright and alert. Physical examination revealed a heart rate of 120 beats per minute, a respiratory rate of 36 breaths per minute, a grade III/VI left basilar systolic murmur, regular rhythm, and good femoral pulse quality. Neurologic examination was normal. A transthoracic echocardiogram (TTE) was performed in right and left lateral recumbency using an 8-MHz probe and a Philips EPIQ ultrasound machine. The right atrium and ventricle were mildly enlarged, with subjective mild right ventricular wall hypertrophy. The tricuspid valve was dysplastic with mild to moderate tricuspid valve regurgitation, and a small secundum ASD was present. Pulsed-wave Doppler showed low-velocity (<1 m/sec) bidirectional flow across the ASD. An agitated saline contrast study was performed to confirm the atrial-level shunt. Three milliliters of physiologic saline (0.9%) were agitated for 1 minute by vigorous transfer between two 3-mL syringes in a closed system without the introduction of additional air into the syringes. Two milliliters of the agitated saline were injected through a saphenous vein catheter. Echocardiographic contrast consistent with intracardiac air microbubbles was detected first in the right atrium, followed by the right ventricle and left atrium, confirming an atrial-level shunt (Video 1). As the intracardiac microbubble artifacts were dissipating, the left ventricular myocardium became regionally hyperechoic, specifically in a left anterior descending and left circumflex coronary artery distribution (midseptum and mitral papillary muscles; Video 2). Figure 1A and B depicts the change in myocardial echogenicity before and after saline contrast echocardiography. This patchy myocardial enhancement persisted for several minutes (Video 3) with slow dissipation over the next 5 minutes. The monitoring electrocardiogram (ECG) showed sinus rhythm without any pathologic ST-T wave changes during the entire TTE.

An ambulatory 24-hour (Holter) ECG monitor was placed after the TTE to evaluate for cardiac arrhythmias as a potential cause of the historical collapse episodes. The resulting ECG recording was normal, with no arrhythmias noted during the 24-hour monitoring period. A neurologic cause of collapse was suspected based on the event description and the normal 24-hour ECG, although paradoxical cerebral embolization could not be ruled out. The dog did not return to the referral hospital for a requested follow-up study but was examined by the primary care veterinarian 2 years later. At that time a routine wellness examination was unremarkable, with no new client complaints noted, and no clinically significant abnormalities were found on a complete blood count or biochemical profile. The collapse episodes continued intermittently but were reported to be reduced in frequency after oral cannabidiol oil was prescribed by the primary veterinarian.

## Discussion

Agitated saline contrast echocardiographic studies are frequently used to aid in the detection of abnormal intracardiac communications such as atrial and ventricular septal defects, patent foramen ovale, or intrapulmonary (arteriovenous) shunts.^1^, ^2^, ^3^, ^4^^,^^6^, ^7^, ^8^ The intravenous administration of agitated saline poses a theoretical risk of air embolization when right-to-left shunts are present, potentially predisposing to tissue ischemia or infarction. There is particular concern for the possibility of myocardial infarction from coronary air embolization.^1^^,^^2^^,^^5^

Depending on the size of the air bubbles, microembolisms can obstruct capillary vascular beds, resulting in tissue ischemia, inflammation, and activation of complement.^5^^,^^9^ In people, paradoxical cerebral air embolisms have been reported following agitated saline study, resulting in transient ischemic attacks or cerebrovascular accidents.^1^^,^^6^^,^^10^ Associated clinical signs have included transient dizziness, weakness, left-sided paresthesia, and migraines.^10^ In rat models, bubbles >45 μm in diameter caused ischemic infarctions in the regions supplied by the right carotid artery.^11^

Standard procedures and protocols for safe agitated saline administration are not yet defined, despite concern for the potential of infarction and ischemia. While size and location of the bubbles appear to be the most important risk factors for infarction, ideal bubble size is difficult to predict and standardize because of dynamic bubble behaviors that can include coalescence, dissolution, and splitting.^2^^,^^6^ An in vitro closed-circuit study showed the potential dangers of bubbles >45 μm.^2^ Generally, lower volumes of air resulted in fewer bubbles >45 μm, but bubbles up to 100 μm could still be created, and injected volumes as low as 0.025 mL resulted in myocardial vessel occlusion in vitro.^2^

Despite the theoretic risk, saline contrast echocardiography is considered a first-line diagnostic tool to identify anomalous shunting because it is simple, effective, and inexpensive when compared with alternative imaging modalities such as computed tomography, cardiovascular magnetic resonance, or nuclear scintigraphy, procedures that usually require animals to be anesthetized.^1^^,^^2^ The frequency of adverse events with saline contrast echocardiography appears to be low, but this has not been quantified. The transient myocardial contrast enhancement that occurred in this dog shortly after the intravenous administration of agitated saline highlights the potential for air to enter the coronary circulation, which could predispose to myocardial infarction. Although no overt air was introduced during agitation and the technique was standardized within our practice, bubble size was not measured and could have been variable. Therefore, the reason for myocardial enhancement in this case remains unknown. Despite the risk of introducing air into the coronary circulation, the use of saline contrast to confirm anomalous coronary artery origins from the pulmonary artery and coronary-cameral fistulae has been reported in humans, given the higher risks associated with more invasive procedures.^8^^,^^12^ Cardiac troponin I measurement before and after the study would have been useful for the identification of myocardial injury associated with the myocardial enhancement in this case; unfortunately, this was not performed. The dog did not show clinical or electrocardiographic signs of myocardial infarction, and no arrhythmias were noted during the 24 hours after the TTE, but this does not rule out the possibility of subclinical cardiomyocyte injury.

## Conclusion

This report of myocardial contrast enhancement after agitated saline contrast study during echocardiography suggests that air microemboli can be inadvertently introduced into the coronary vasculature in the presence of a right-to-left shunt. Studies evaluating the safety profile of saline contrast echocardiography in dogs are indicated.

## Ethics Statement

The authors declare that the work described has been carried out in accordance with the following guidelines: the clinical care provided to the dog of this case report was in accordance with the ethical practice guidelines of the hospital.

## Consent Statement

Complete written informed consent was obtained from the patient (or appropriate parent, guardian, or power of attorney) for the publication of this study and accompanying images.

## Funding Statement

The authors declare that this report did not receive any specific grant from funding agencies in the public, commercial, or not-for-profit sectors.

## Disclosure Statement

The authors report no conflict of interest.

## Supplementary Data

Video 1 Two-dimensional TTE in the right parasternal 4-chamber long-axis view showing microbubble echocardiographic artifact in the right atrium followed by the right ventricle and left atrium, confirming a right-to-left atrial-level shunt.

Video 2 Two-dimensional TTE in the right parasternal 4-chamber long-axis view showing left ventricular myocardial enhancement during dissipation of intracardiac chamber echocardiographic contrast.

Video 3 Two-dimensional TTE in the right parasternal 4-chamber short-axis view showing left ventricular myocardial enhancement after clearing of intracardiac chamber echocardiographic contrast.
